# The activities of daily living partially mediate the relationship between rapid eye movement sleep behavior disorder and depressive symptoms in Parkinson's disease

**DOI:** 10.3389/fneur.2024.1357721

**Published:** 2024-07-22

**Authors:** QiuShuang Wang, ShuangShuang Zheng, Bian Jing, Yi Sun, Wei Qian, ZiXuan Zhao, HuaShuo Zhao

**Affiliations:** ^1^Department of Epidemiology and Biostatistics, School of Public Health, Xuzhou Medical University, Xuzhou, Jiangsu, China; ^2^Department of Rehabilitation Medicine, The 334 Affiliated Hospital of Nanchang University, Nanchang, China; ^3^Department of Medical Records, The Affiliated Hospital of Xuzhou Medical University, Xuzhou, Jiangsu, China; ^4^School of Health Economics and Management, Nanjing University of Traditional Chinese Medicine, Nanjing, Jiangsu, China

**Keywords:** Parkinson's disease, rapid eye movement sleep behavior disorder, depression, activities of daily living, longitudinal mediation

## Abstract

**Objective:**

A longitudinal study was conducted to investigate whether rapid eye movement sleep behavior disorder affect depression in patients with Parkinson's disease through activities of daily living.

**Methods:**

A total of 387 Parkinson's disease patients' six-year follow-up data (one follow-up per year) were obtained from the Parkinson's Progression Markers Initiative. To allow causal effects to manifest, this study increased the lag period and divided the data from the six follow-ups into two groups: wave 1 (wave refers to time points), wave 3, and wave 5 as one group, and wave 2, wave 4, and wave6 as the other group. The time interval between two time points in each group was two years. To comprehensively and deeply analyze the dynamic relationships between variables, accurately infer causal relationships, control for individual differences, and detect the stability of these relationships, this study constructed the fixed effects cross-lagged panel model (CLPM), the random effects CLPM (RE-CLPM) model, and the Equating CLPM and Equating RE-CLPM models with applied restriction conditions. Additionally, a reverse path was added to verify the reverse prediction effect. The most suitable data analysis model was selected to explore the relationships between the study variables. Furthermore, the longitudinal mediating effect of daily living activities between rapid eye movement sleep behavior disorder and depression was investigated.

**Results:**

In the models, Equating cross-lagged panel model was the best. The lag effect was positive and significant. In wave 1, 3, 5, activities of daily living mediated 11.82% on the path from rapid eye movement sleep behavior disorder to depression; in wave 2, 4, 6, it mediated 13.13%. Therefore, attention should be paid to the treatment of activities of daily living.

**Conclusion:**

Longitudinal changes in activities of daily living have indirect effects on the relationship between rapid eye movement sleep behavior disorder and depression, which highlights the importance of changes in activities of daily living ability in Parkinson's disease patients with rapid eye movement sleep behavior disorder.

## 1 Introduction

Parkinson's disease (PD) is a progressive neurodegenerative disorder that results from the loss of dopamine-producing nerve cells in the substantia nigra. The disease is characterized by bradykinesia, tremors at rest, and postural instability, which are its main features. However, non-motor symptoms such as depression, sleep disorders, constipation, loss of smell, anxiety, autonomic, and cognitive dysfunction may appear several years before motor symptoms in PD patients (1–3). Before the clinical diagnosis of PD, about 90.3% of patients experience prodromal non-motor symptoms (4), although these symptoms can also occur in the elderly without PD. Nonetheless, the prevalence of non-motor symptoms is significantly higher in the PD group than in the healthy population (5, 6). Depression, for example, is one of the most common non-motor symptoms and affects 10%-90% of PD patients (7–9). Depression is a common mood disorder characterized by persistent, severe, and prolonged feelings of sadness, loss of interest or pleasure, self-deprecation, lack of energy, and a decreased interest and participation in daily activities. Depression can affect an individual's thinking, behavior, emotions, and physical health. Although conventional antidepressants are commonly used to treat depression in PD patients, their benefits are moderate and the results of clinical trials are uncertain (10).

Approximately 80% of individuals with PD will experience sleep disturbances (11). These disturbances may include insomnia, restless leg syndrome, rapid eye movement sleep behavior disorder (RBD), excessive daytime sleepiness, and other symptoms (12, 13). RBD is a sleep disorder characterized by vivid dreams and physical activity during rapid eye movement sleep. It can be classified as idiopathic RBD or symptomatic RBD (14). Idiopathic RBD refers to RBD without evidence of neurodegeneration and has a high predictive value, with around 50% of individuals with idiopathic RBD developing a neurodegenerative disease within 10 years (15, 16). RBD has also been associated with depression. Neikrug et al. (17) conducted a cross-sectional study and found that PD patients with RBD exhibit more non-motor symptoms, including increased sleep complaints, higher levels of depressive symptoms, lower quality of life, poorer cognitive abilities, and increased fatigue. Cui et al. (18) conducted a cross-sectional survey of 403 PD patients using multivariate logistic analysis to investigate the prevalence and risk factors of depression and anxiety in Parkinson's patients. They found that more severe motor function, motor disturbances, poorer sleep quality, and anxiety are risk factors for depression in PD. Studies have indicated that insomnia often precedes the onset of depression and that individuals with chronic insomnia are at a higher risk of developing depression due to disruptions in the hypothalamic-pituitary-adrenal axis (19, 20). The close relationship between RBD and depression has also been confirmed in animal studies. Animal models of RBD based on α-synuclein disease in mice have demonstrated that RBD is not static and can progress to include Parkinson's motor dysfunction, depression-like disorders, and olfactory dysfunction, among others (21).

Activities of daily living (ADLs) are often perceived as routine tasks, but they are intricate and multidimensional, reflecting both motor and cognitive abilities (22). As PD progresses, the performance of ADLs can become compromised, leading to symptoms like tremors, pain, and disruptions to one's daily rhythm. These challenges can contribute to a psychological burden, causing individuals to withdraw and experience emotional instability, irritability, and worsened depression. Emotional symptoms can significantly impact the quality of life and ability to perform ADLs in older individuals with chronic conditions (23, 24). Numerous studies have demonstrated a positive association between sleep quality and ADLs (25, 26).

The aforementioned research suggests a potential link between ADLs, depression, and RBD. While previous studies have explored ADLs as an outcome variable, this study takes a different approach by considering ADLs as an intermediary variable. It hypothesizes that RBD may affect the depressive state of Parkinson's patients over time through its impact on ADLs. The application of longitudinal mediating models is widespread in psychology and education, but their use in medical research has been limited. Previous studies have utilized the random intercept cross-lagged panel model (RI-CLPM) to explore the relationships among sleep quality, depressive symptoms, and cognitive function, concluding that sleep disorders partially mediate the relationship between cognitive abilities and depressive symptoms (27). Additionally, research employing progressive autoregressive mediation models has investigated the impact and relationships between depression, anxiety, and autonomic dysfunction, finding that in patients with PD, autonomic dysfunction has a longitudinal mediating effect on the onset and exacerbation of anxiety and depression through ADLs (28). In this study, a longitudinal mediation model was constructed to investigate how sleep and daily living activities influence depressive symptoms in patients with PD.

## 2 Methods

### 2.1 Data and sample

**Figure 1:**
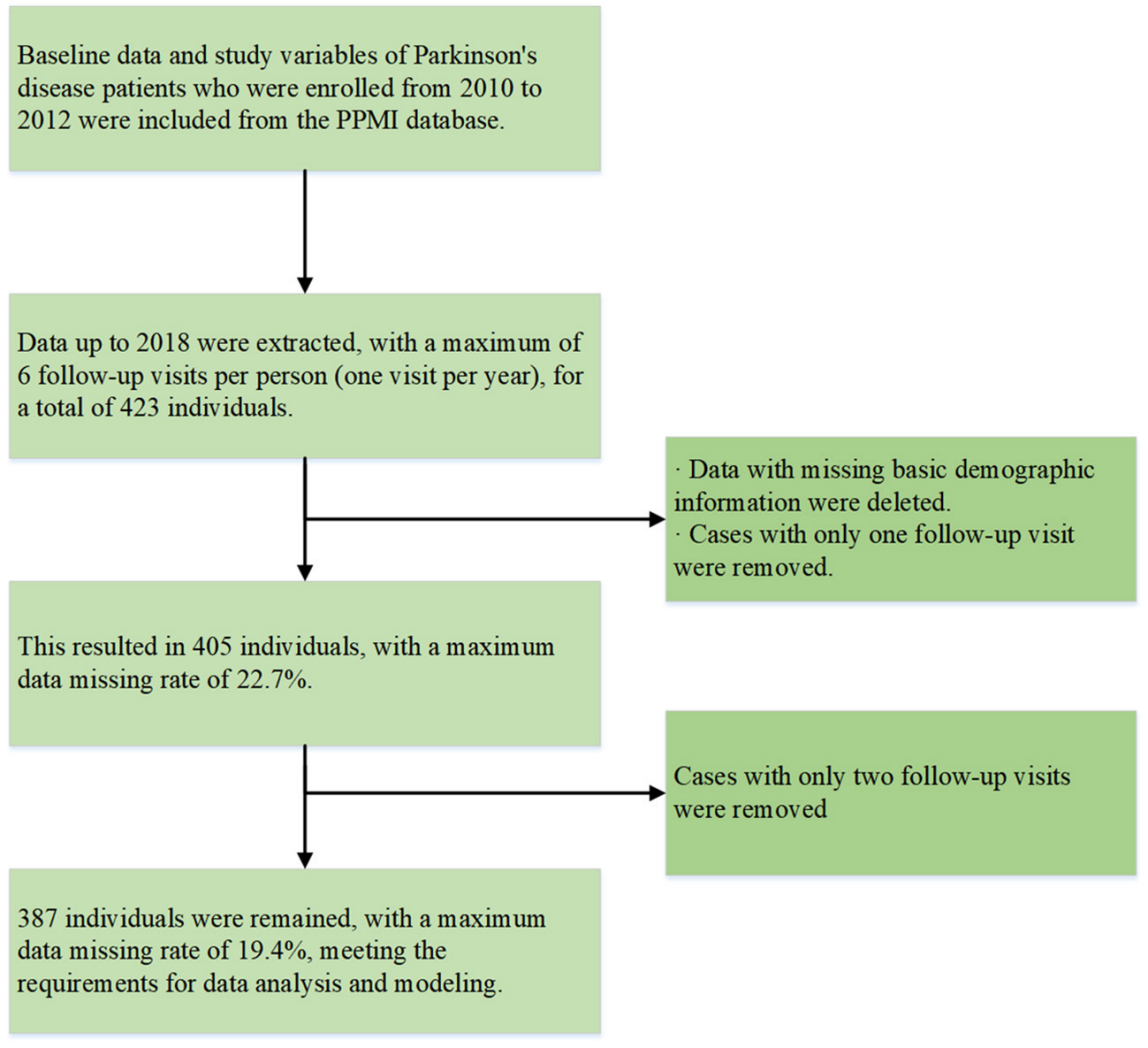
Sample selection.

In 2010, The Michael J. Fox Foundation and a core group of academic scientists and industry partners launched the Parkinson's Progression Markers Initiative (PPMI), which aims to investigate much-needed biomarkers for the onset and progression of PD. Data used in the preparation of this article were obtained from the PPMI database (www.ppmi-info.org/access-data-specimens/download-data), RRID:SCR_006431. For up-to-date information on the study, visit www.ppmi-info.org. Follow-up data from 2010 to 2018 in PPMI, including individuals diagnosed with PD, were selected. Those missing basic demographic information or with only 1–2 follow-up data points were excluded. The specific sample inclusion criteria are shown in Figure 1, with our final sample size maintained at 387 participants. None of our participants received treatment at baseline, but underwent confirmative assessments, including clinical and cognitive evaluations, imaging examinations, and biological sampling, which were approved by the local participant Central Institutional Review Board. All participants provided written informed consent prior to enrollment.

### 2.2 Measurements

Rapid Eye Movement Sleep Behavior Disorder (RBD) was measured using the Rapid Eye Movement Sleep Behavior Disorder Screening Questionnaire (RBDSQ), which consists of 10 items (29). Items 1–4 inquire about the frequency, content, and relationship between dreams and behavior. Item 5 assesses self-injury and injury to others. Item 6 is subdivided into four sub-items that evaluate specific instances of abnormal movements during sleep, such as sleep talking, sudden limb movements, complex movements, etc. Items 7 and 8 pertain to nocturnal awakenings, item 9 evaluates overall sleep quality, and item 10 focuses on central nervous system diseases. The total score on the RBDSQ is 13 points, with a cut-off value of 5 points for the general population (29) and 6 points for individuals with PD (30). The higher the RBDSQ score, the greater the likelihood of RBD occurrence.

In our assessment of activities of daily living (ADLs) in Parkinson's patients, we utilized the second part of the Unified Parkinson's Disease Rating Scale (UPDRSII). This inventory comprises 13 items and covers fundamental ADLs, including eating, dressing, hygiene, walking, hobbies, etc. The total score is calculated as the sum of these 13 items, resulting in a maximum score of 52 points. A higher score indicates more severe impairment in ADLs.

Depression levels were evaluated using a shortened version of the Geriatric Depression Scale (GDS), specifically the GDS-15. This scale consists of 15 items that assess depression symptoms, reduced activity, irritability, withdrawal from social interactions, negative thoughts about the past, present, and future. The overall GDS score ranges from 0 to 15, with higher scores indicating a greater burden of depression. A score of 5 or above suggests the presence of depression. The GDS-15 demonstrates good reliability and validity when used with the elderly population (31). The measurements of the variables (RBDSQ, UPDRSII, GDS) were conducted at different times throughout each year for each individual, but for each individual, the measurements were conducted ~1 year apart.

### 2.3 Statistical analysis

#### 2.3.1 Mediation analysis of the cross-lagged panel model and random effect cross-lagged panel model

When conducting mediating analyses, we prefer to use longitudinal data rather than cross-sectional data models. The cross-lagged panel mediation (CLPM) method (32) is preferred as it has a stronger inference of causality and can reduce parameter bias. CLPM can be classified into two types: fixed effect CLPM and random effect CLPM (RE-CLPM). One limitation of fixed effect CLPM is that the effect is assumed to be fixed among individuals. However, this assumption may not hold in practice, which may lead to biased parameter estimation and misleading statistical inference. RE-CLPM permits the effects in the model to be random and can account for the potential individual variability of the effects and assess the covariant relationships that such variability may produce (33). However, RE-CLPM also has its limitations. Firstly, due to random effects, RE-CLPM can only use information criteria to evaluate model fitting and cannot use common model fitting indicators such as Chi-square test statistics, Approximate Root Mean Square Error (RMSEA), Comparative Fit Index (CFI), and Tuck-Lewis Index (TLI). Secondly, it can only be applied if the dependent variable is continuous. When there are random effects in our population, RE-CLPM is more suitable for our model. However, in the actual application process, we do not know if there are real random effects in our model. Therefore, we first construct a fixed effect model, then release the random effects in the model to construct a random effect model, and finally compare the two models to select the best one (33). The CLPM assumes that the prospective relationships between variables remain stable over time. In order to achieve this, we imposed equality constraints on the autoregressive and cross-hysteresis paths, assuming that these effects remain constant over time, which resulted in Equating CLPM and Equating RE-CLPM.

**Figure 2:**
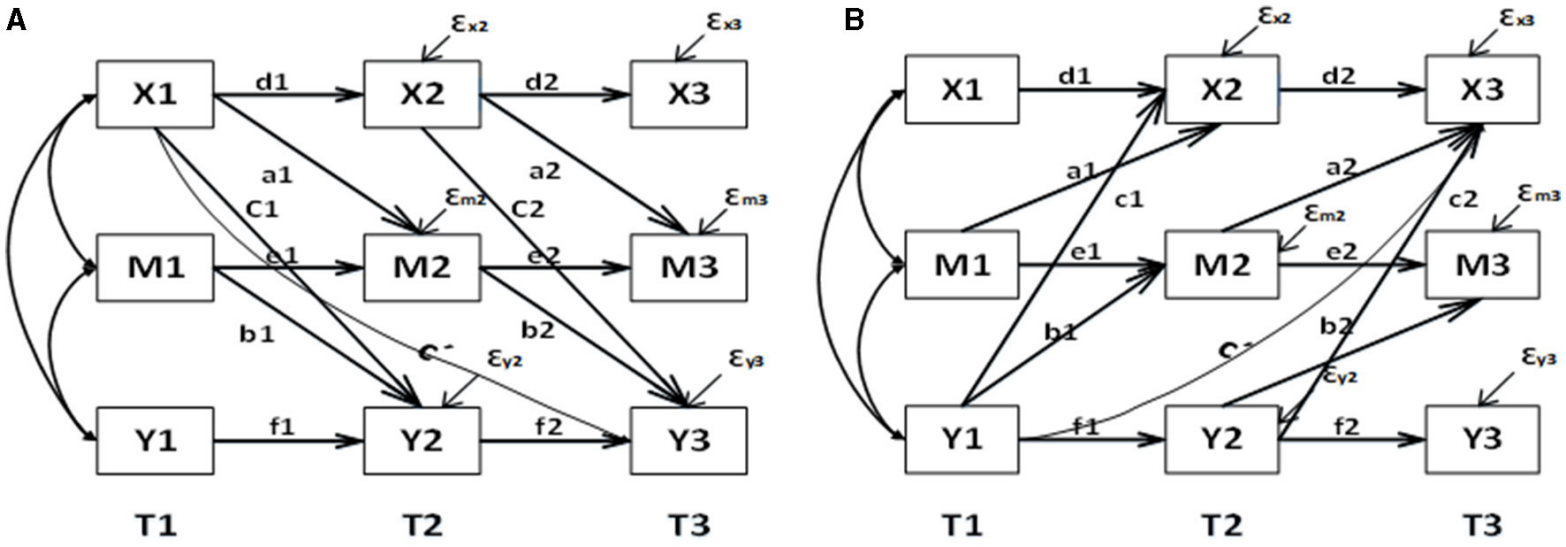
Mediation model and reverse mediation mode based on CLPM longitudinal data. **(A)** For the forward model and **(B)** for the reverse model. d1, d2, e1, e2, f1, f2 represent autoregressive coefficients; a1, a2, b1, b2, C', c1, c2 represent cross-lagged coefficients, with C' indicating the direct effect; a1*b2 indicates the longitudinal mediation effect.

Figure 2A illustrates the relationship between three waves of variables: X (RBD), M (ADLs), and Y (depression). The three time points in our follow-up are denoted as T1, T2, and T3. To investigate the mediating role of ADLs between RBD and depression, we designed a path from RBD at a previous time point to ADLs at a later time point, and another path from ADLs at a previous time point to depression at a later time point. Additionally, to examine whether the mediating role is partial or full, we increased the regression pathway from RBD at T1 to depression at T3. We also included paths in the opposite direction to test the predictive effect (Figure 2B). In longitudinal studies exploring mediating effects, it is crucial to carefully consider the choice of time interval. Prior to conducting the experiment, we need to assess the time interval between the independent variable and the mediating variable, as well as the mediating variable's influence on the dependent variable. As mentioned earlier, non-motor symptoms may manifest several years before motor symptoms. However, since we are using data from an existing database, we do not have control over the time interval. Therefore, we optimized our experimental design by dividing the six-wave data into two groups for model verification. Group 1 consists of wave 1, wave 3, and wave 5, while group 2 includes wave 2, wave 4, and wave 6. It is important to note that during the modeling process, we had to fix the mediation-related autoregressive effects in the model due to convergence issues when releasing them as random effects.

In this model, the total effect (OTE) comprises the total indirect effect (OIE) and the total direct effect (ODE). Our primary focus lies on the OIE, as it indicates the extent to which the independent variable influences the dependent variable through mediations. In the case of fixed effects CLPM, we employ the coefficient product method to calculate the indirect effect (34). As for RE-CLPM, the mean of the intermediate effect is obtained by multiplying the means (a and b) of the random coefficients a and b and adding the covariance of a and b (33). Similarly, the mean of the direct effect is determined by the mean c' of the random coefficients c'. To quantify the effect of mediation, we utilize the intermediation percentage formula, which involves dividing the indirect effect by the total effect.

#### 2.3.2 Model fitting index and comparison of each model

For the missing data, a multiple interpolation method was used to fill in the data, and modeling was then conducted. Non-standardized data were utilized in the analysis, as standardized data may lead to inaccurate parameter estimates, standard errors, and goodness-of-fit indices (30, 35, 36). To assess the CLPM model, RMSEA, CFI, and TLI values were relied upon, as the χ^2^ goodness-of-fit statistic can be overly sensitive for large sample sizes and was therefore not employed.RMSEA is a measure of how well a statistical model fits the population's covariance matrix. It takes into account the model complexity by including a penalty for the number of estimated parameters. RMSEA value below 0.05 indicates a good fit to the data, while a value between 0.05 and 0.08 suggests a suitable fit (37). CFI compares the fit of a target model to an independent baseline model, which assumes no relationships among the variables. It is a measure of the improvement in fit provided by the target model relative to the baseline model. TLI, also known as the non-normed fit index, compares the fit of a target model to a baseline model, similar to the CFI. However, it also includes a penalty for model complexity. For CFI and TLI, a value above 0.90 is considered acceptable, and a value exceeding 0.95 indicates a good fit (38). When comparing CLPM and RE-CLPM, two aspects need to be examined. Firstly, we consider the information standards such as the Akaike Information Criterion (AIC), Bayesian Information Criterion (BIC), and adjusted BIC (ABIC). A smaller value signifies a better-fitting model. Additionally, meaningful differences between the two sets of results are checked. If no significant differences are found, it is reasonable to report the CLPM results.

To implement the necessary model and conduct the analysis, Mplus 8.9 is utilized. Descriptive analysis and plotting are performed using SPSS version 25.0 and R (version 4.2.3). The test level is set at a *p*-value of 0.05.

## 3 Results

### 3.1 Descriptive statistics

The data do not meet the normal distribution in all groups, so the median and interquartile range [M(P25~P75)] were used for the description of the data. For comparisons between two groups, we employed the Mann-Whitney U test with the Z statistic. For comparisons involving more than two groups, we used the Kruskal-Wallis rank sum test with the H statistic. The results are shown in Table 1. The subjects of this study were primarily Parkinson's patients over 56 years old (70.80%, 274/387), with 256 males (66.15%) and 131 females (33.85%). There were 323 individuals with ≥13 years of education, but only 2 had more than 23 years of education. The majority of our sample were white people (92.25%, 357/387), and most did not have a family history of the disease (74.94%, 290/387). The Hoehn and Yahr staging was mainly concentrated in stages 1 and 2, with only two individuals in stages 3–5. Notably, GDS scores did not significantly differ by demographic characteristics (p > 0.05), indicating that including these factors as covariates in the model was unnecessary.

**Table 1:** Geriatric depression scale scores and demographic characteristics at baseline (*N* = 387).

**Variables**	**Classification (*N*)**	**M (P25–P75)**	**Z/H value**	***p*-value**
Age (years old)	< 56 (113)	2.000 (1.000–3.000)	1.097	0.578
	56–65 (134)	1.000 (0.750–3.000)		
	>65 (140)	2.000 (1.000–3.000)		
Gender	Male (256)	2.000 (1.000–3.000)	−0.210	0.834
	Female (131)	2.000 (1.000–3.000)		
Education (years)	< 13years (64)	2.000 (0.000–3.750)	−0.543	0.587
	≥13years (323)	2.000 (1.000–3.000)		
Race	White (357)	2.000 (1.000–3.000)	1.779	0.075
	Non-white (30)	2.000 (1.000–5.000)		
Family history	Direct relative (52)	2.000 (0.000–3.000)	2.125	0.346
	Indirect relative (45)	2.000 (1.000–3.500)		
	NO (290)	2.000 (1.000–3.000)		
Hoehn-Yahr	Stage 1 (175)	1.000 (0.000–3.000)	1.449	0.147
	Stage 2–5 (212)	2.000 (1.000–3.000)		

M (P25–P75): Median (25th to 75th percentile); the values in the third column are GDS scale scores; Significant at p < 0.05.

**Figure 3:**
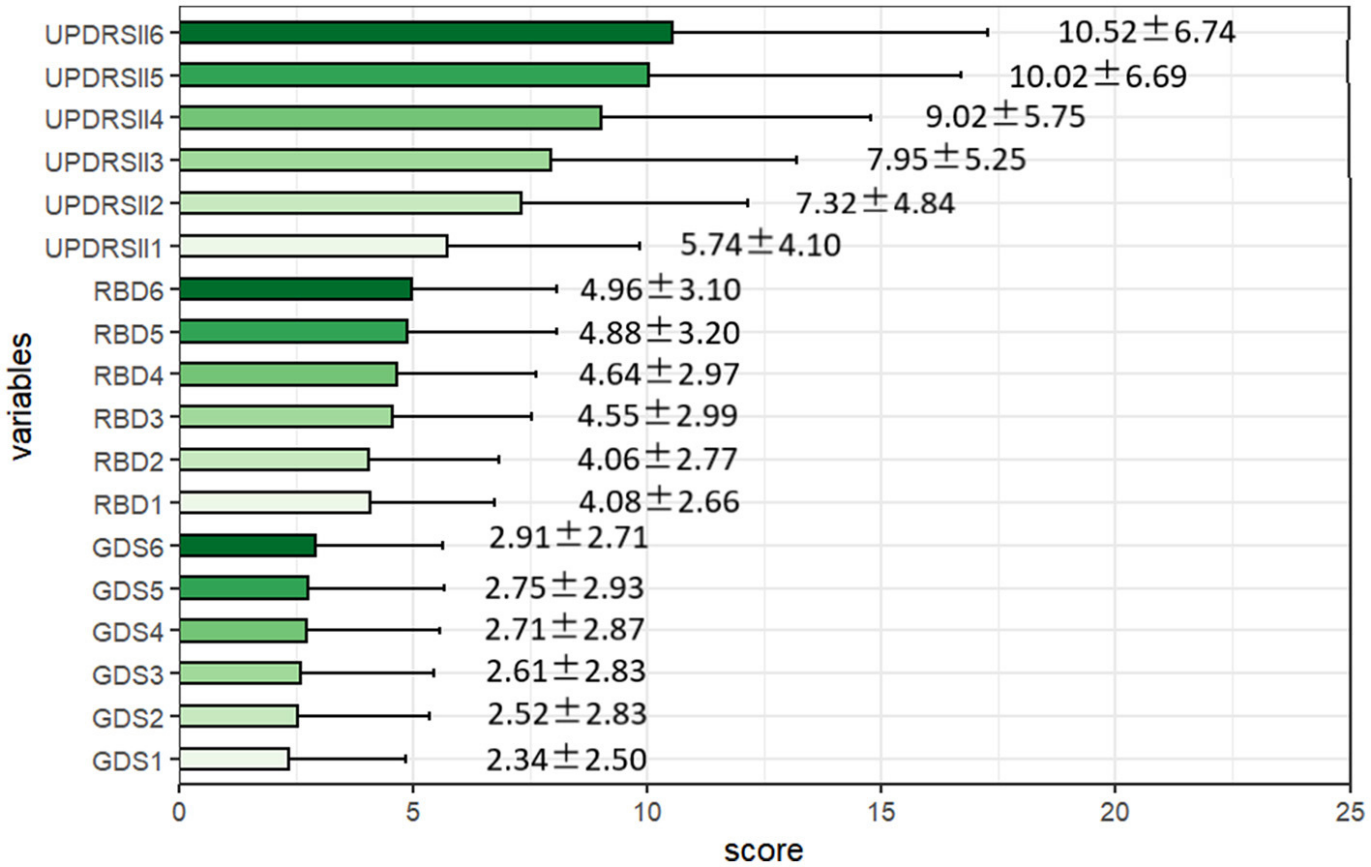
Descriptive characteristics of the rapid-eye-movement sleep behavior disorder, depression, and activities of daily living scores over the 6-month follow-ups. RBD, the Rapid-eye-movement Sleep Behavior Disorder; GDS, Geriatric Depression Scale; UPDRS-II, The second part of the Unified PD Rating Scale.

In Figure 3, we can see that the scores for GDS, RBD, and UPDRSII gradually increased over the six follow-up visits, indicating that the conditions of RBD and depression gradually worsened over time, while the ability in ADLs declined over time.

**Figure 4:**
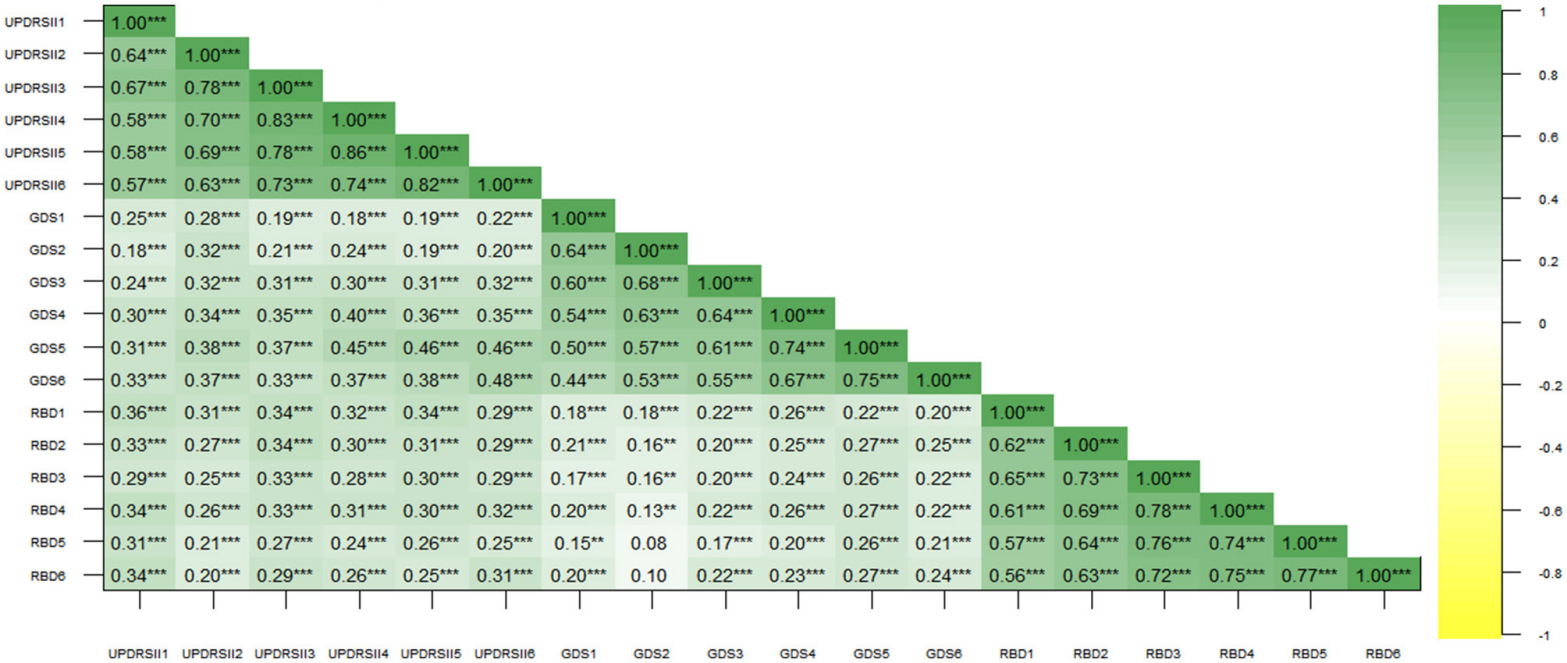
Correlation analysis of Rapid-Eye-Movement Sleep Behavior Disorder (RBD), Geriatric Depression Scale (GDS), and Unified Parkinson's Disease Rating Scale (UPDRS-II) scores. Numbers in the graph represent correlation coefficients. ** represents *p* < 0.01; *** represents *p* < 0.001.

Moreover, in Figure 4, we described the pairwise correlations between RBD, ADLs, and depression in PD patients through Pearson correlation analysis, revealing a generally significant positive correlation between each pair. This also corroborates our hypothesis regarding the relationships among the three variables, highlighting the necessity of exploring their interconnections.

### 3.2 Mediation analysis of CLPM and RE-CLPM

Firstly, the CLPM and Equating CLPM were constructed as depicted in Figure 2. Subsequently, the random effects were released to create the RE-CLPM and Equating RE-CLPM. In Tables 2, 3, better fitting indices for Equating CLPM compared to CLPM were observed. When comparing Equating RE-CLPM and Equating CLPM, it was found that the C1 and C2 paths in Equating CLPM were significant. Additionally, the AIC, BIC, and ABIC values were not different between the two models, and the standard error (SE) in Equating CLPM was smaller than in Equating RE-CLPM. Taking into account the fitting indices, significance of path coefficients, and standard deviation, we concluded that Equating CLPM was the superior model among the four.

**Table 2:** Fit indices of structured equation models for mediation analysis of cross-lagged panel model (wave 1, wave 3, wave 5).

**Effect**	**CLPM**			**RE-CLPM**			**Equating CLPM**			**Equating RE-CLPM**		
	**Beta**	**se**	***p*** **-value**	**Beta**	**se**	***p*** **-value**	**Beta**	**se**	***p*** **-value**	**Beta**	**se**	***p*** **-value**
a1	0.238	0.086	0.005	0.209	0.080	0.009	0.161	0.054	0.003	0.191	0.055	< 0.001
a2	0.096	0.076	0.205	0.116	0.071	0.102	0.161	0.054	0.003	0.191	0.055	< 0.001
b1	0.048	0.030	0.102	0.033	0.029	0.260	0.080	0.022	< 0.001	0.078	0.030	0.010
b2	0.105	0.030	0.001	0.075	0.028	0.008	0.080	0.022	< 0.001	0.078	0.030	0.010
C'	−0.082	0.057	0.151	−0.047	0.043	0.268	−0.055	0.050	0.270	−0.039	0.050	0.434
a1^*^b2	0.025	0.012	0.036	0.018	0.011	0.110	0.013	0.006	0.026	0.024	0.009	0.006
C1	0.094	0.047	0.045	0.099	0.050	0.050	0.113	0.035	0.001	0.096	0.050	0.057
C2	0.143	0.056	0.010	0.077	0.044	0.082	0.113	0.035	0.001	0.096	0.050	0.057
RMSEA	0.083						0.077					
CFI	0.977						0.971					
TLI	0.946						0.953					
AIC	16,977.938			167,700.048			16,980.708			16,867.441		
BIC	17,136.275			16,983.803			17,115.294			17,033.695		
ABIC	17,009.359			16,812.467			17,007.416			16,900.433		

Significant at p < 0.05; RE-CLPM, random effects cross-lagged panel model; CLPM, cross-lagged panel model;beta represents the strength and direction of cross-lagged effects, autoregressive effects, or mediation effects; se, standard error; RMSEA, Root Mean Square Error of Approximation; CFI, Comparative Fit Index; TLI, Tucker-Lewis Index; AIC, Akaike Information Criterion; BIC, Bayesian Information Criterion; ABIC, Sample-Size Adjusted Bayesian Information Criterion.

**Table 3:** Fit indices of structured equation models for mediation analysis of cross-lagged panel model (wave 2, wave 4, wave 6).

**Effect**	**CLPM**			**RE-CLPM**			**Equating CLPM**			**Equating RE-CLPM**		
	**Beta**	**se**	***p*** **-value**	**Beta**	**se**	***p*** **-value**	**Beta**	**se**	***p*** **-value**	**Beta**	**se**	***p*** **-value**
a1	0.246	0.081	0.003	0.236	0.079	0.003	0.237	0.054	< 0.001	0.248	0.057	< 0.001
a2	0.225	0.078	0.004	0.184	0.067	0.006	0.237	0.054	< 0.001	0.248	0.057	< 0.001
b1	0.063	0.025	0.011	0.054	0.027	0.048	0.057	0.014	< 0.001	0.063	0.244	0.797
b2	0.054	0.017	0.001	0.054	0.014	< 0.001	0.057	0.014	< 0.001	0.063	0.244	0.797
C'	0.060	0.044	0.173	0.049	0.044	0.260	−0.012	0.042	0.778	−0.017	0.123	0.889
a1^*^b2	0.013	0.006	0.032	0.039	0.017	0.024	0.013	0.004	0.003	0.016	0.043	0.706
C1	0.134	0.045	0.003	0.090	0.043	0.034	0.073	0.032	0.021	0.069	0.181	0.704
C2	−0.015	0.041	0.712	−0.024	0.041	0.567	0.073	0.032	0.021	0.069	0.181	0.704
RMSEA	0.105						0.089					
CFI	0.966						0.965					
TLI	0.920						0.942					
AIC	17,117.256			17,012.013			17,113.853			17,114.859		
BIC	17,275.593			17,225.768			17,248.440			17,281.113		
ABIC	17,148.677			17,054.432			17,140.561			17,147.851		

Significant at p < 0.05; RE-CLPM, random effects cross-lagged panel model; CLPM, cross-lagged panel model; beta represents the strength and direction of cross-lagged effects, autoregressive effects, or mediation effects; se, standard error; RMSEA, Root Mean Square Error of Approximation; CFI, Comparative Fit Index; TLI, Tucker-Lewis Index; AIC, Akaike Information Criterion; BIC, Bayesian Information Criterion; ABIC, Sample-Size Adjusted Bayesian Information Criterion.

In Table 4, the mediation effects in Equating CLPM for the two data sets are summarized. Four paths were observed between X1 → Y5 and X2 → Y4, respectively, consisting of one direct path and three indirect paths: R1, R2, and R3. The main focus was on R1, which demonstrated the effect of RBD at time T1 on Depression at time T3 through ADLs at time T2. R1 pathway was particularly emphasized, with total effects of 0.110 and 0.099, both significant at 0.013 (*p* < 0.05). Although not very large, ADLs accounted for 11.82% in waves 1, 3, and 5, and 13.13% in waves 2, 4, and 6. Therefore, we can conclude that ADLs partially mediate the relationship between RBD and Depression.

**Table 4:** Mediating effect of equating cross-lagged panel model.

**Effect**	**Equating CLPM (wave 1, wave 3, wave 5)**				**Equating CLPM (wave 2, wave 4, wave 6)**			
	**Road**	**Beta**	**se**	***p*** **-value**	**Road**	**Beta**	**se**	***p*** **-value**
ODE	X1 → Y5	−0.055	0.050	0.270	X2 → Y6	−0.012	0.042	0.778
OIE	R1:X1 → M3 → Y5	0.013	0.006	0.026	R1:X2 → M4 → Y6	0.013	0.004	0.003
	R2:X1 → X3 → Y5	0.088	0.028	0.001	R2:X2 → X4 → Y6	0.056	0.025	0.024
	R3:X1 → Y3 → Y5	0.065	0.019	0.001	R3:X2 → Y4 → Y6	0.042	0.018	0.020
OTE		0.110	0.052	0.033		0.099	0.041	0.016
R1/OTE	11.82%				13.13%			

OTE, The overall total effect; OIE, the overall indirect effect; ODE, The overall direct effect; Significant at p < 0.05.

**Figure 5:**
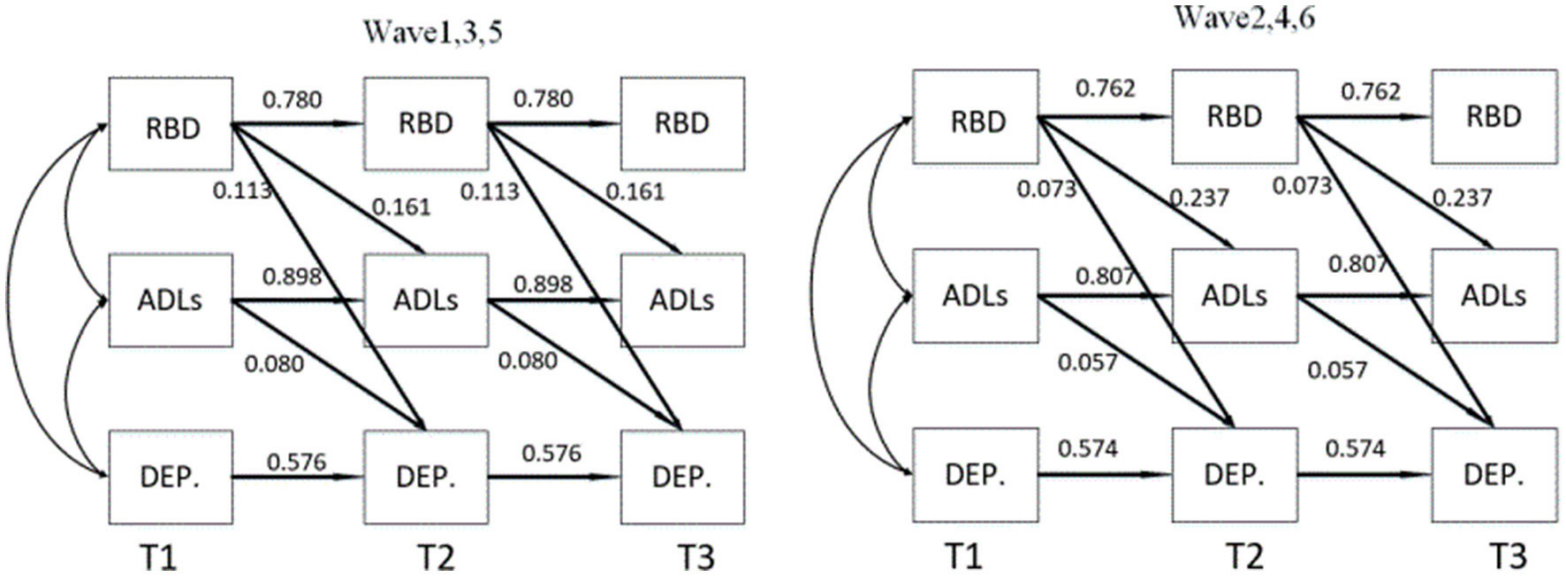
The mediating effect of activities of daily living on the relationship between Rapid Eye Movement Sleep Behavior Disorder and depression. ADLs, Activities of daily living; RBD, Rapid Eye Movement Sleep Behavior Disorder; DEP., Depression; T1, T2 and T3 were the duration of the three follow-up visits.

To test the predictive effect in the opposite direction, we employed Equating CLPM for modeling, as shown in Table 5. However, the path coefficient of the mediation model was not significant, further confirming the previous findings and supporting our hypothesis. Figure 5 provides an overall diagram of our model. To simplify the plot, only significant path coefficients are included.

**Table 5:** Fit indices of structured equation models for mediation analysis of inverse equating cross-lagged panel model.

**Effect**	**Inverse equating CLPM (wave 1, 3, 5)**			**Inverse equating CLPM (wave2, 4, 6)**		
	**Beta**	**se**	***P*** **-value**	**Beta**	**se**	***P*** **-value**
a1	0.018	0.020	0.351	0.021	0.016	0.203
a2	0.018	0.020	0.351	0.021	0.016	0.203
b1	0.149	0.067	0.027	0.103	0.063	0.100
b2	0.149	0.067	0.027	0.103	0.063	0.100
C'	−0.013	0.052	0.801	−0.042	0.036	0.252
a1^*^b2	0.003	0.004	0.440	0.002	0.002	0.369
C1	0.041	0.035	0.245	0.041	0.036	0.252
C2	0.041	0.035	0.245	0.041	0.036	0.252
RMSEA	0.102			0.110		
CFI	0.950			0.947		
TLI	0.918			0.912		
AIC	17,015.209			17,145.416		
BIC	17,149.796			17,280.003		
ABIC	17,041.917			17,172.124		

Significant at p < 0.05; RE-CLPM, random effects cross-lagged panel model; CLPM, cross-lagged panel model; beta represents the strength and direction of cross-lagged effects, autoregressive effects, or mediation effects; se, standard error; RMSEA, Root Mean Square Error of Approximation; CFI, Comparative Fit Index; TLI, Tucker-Lewis Index; AIC, Akaike Information Criterion; BIC, Bayesian Information Criterion; ABIC, Sample-Size Adjusted Bayesian Information Criterion.

## 4 Discussion

As depicted in Table 4, the analysis revealed that the total effect value amounts to 0.110 and 0.099 consecutively. It was evident that ADLs partially act as a mediator between RBD and Depression, exhibiting indirect effects of 11.82% and 13.13%, respectively, although these effects are not notably substantial, they are both positive and statistically significant. This implies that the decline in RBD impacts ADLs, while the deterioration of ADLs exacerbates depressive symptoms in individuals with PD. The relationship between RBD and depression may involve multiple “mediation chains,” such as orthostatic hypotension, drooling, swallowing difficulties, and constipation (39). The mediating role of ADLs is also one of the many steps between RBD and depression, and it warrants our attention. In our study, the reverse path was unable to confirm that depression impacts activities of daily living, despite this being supported by previous research (23).

Neuropathological studies have found that in patients with PD, there is not only degeneration of dopaminergic neurons in the substantia nigra striatum leading to motor symptoms but also structural and functional impairments in the locus coeruleus and the dorsal raphe nucleus. These impairments affect the limbic system and its connectivity with the cortex, thereby causing symptoms of mood disorders, depression, memory, and cognitive impairments (40, 41). In patients with PD, those suffering from depression show a significant reduction in the levels of dopamine transporters in the limbic system (including the cingulate gyrus, frontal lobe, locus coeruleus, and dorsal raphe nucleus) early in the course of the disease. Furthermore, the extent of this reduction is positively correlated with the severity of depression. Structural changes can also be detected in the locus coeruleus and subcoeruleus in patients with PD, and these changes are particularly associated with increased muscle tone during REM sleep (42). Therefore, the potential mechanisms linking RBD and depression, particularly in relation to these brain regions, warrant further experimental investigation.

It is essential to note that depression is often considered an integral part of the disease and can be ameliorated and treated using both pharmacological and non-pharmacological approaches. During drug treatment, patients should focus on fostering self-confidence, relaxation, maintaining optimism, redirecting attention, ensuring sufficient sleep, engaging in physical exercise, and developing personal hobbies, etc. Given the partial mediation of ADLs, special attention should be given to addressing the treatment of ADLs in PD patients to alleviate depressive symptoms. As we are aware, UPDRS Part II serves as an assessment tool for evaluating the daily functioning abilities of individuals with PD. Its focus extends beyond mere behaviors and encompasses the individual's ability to carry out activities of daily living. Consequently, the current treatment approach primarily involves training and enhancing their functional capacities. Physical therapy (PT) is commonly employed for ADLs treatment, aiming to maximize the functionality of PD patients through exercise rehabilitation. PT primarily concentrates on improving upper limb function, maintaining posture, enhancing balance, ameliorating gait abnormalities, facilitating transfers, and increasing overall physical activity (43). Moreover, innovative treatment techniques incorporating artificial intelligence, virtual reality, and Motorimagery techniques coupled with PT imagery have demonstrated clinically significant effects on motor function, balance, and ADLs (44).

There are several limitations to the current study. Firstly, subjective reports of RBD symptoms may be influenced by factors unrelated to RBD, such as injury risk, the presence and quality of sleep of bed partners, and recall of RBD-related behaviors (45). Secondly, demographic data were only available at baseline, and relevant variables were not collected at each follow-up. Confounding factors such as age and sex were not adjusted for when building the model, as no significant effects of demographic variables (age, sex, education, race, and family history) on GDS scores were found. Thirdly, although the PPMI database represents a large global cohort, the majority of participants are from Europe and North America (46), making study population predominantly white people. This demographic limitation may restrict the generalizability of the findings. In addition, while the sample size and choice of waves for analysis meet the requirements for establishing a CLPM model (34), larger samples are preferable for drawing more accurate conclusions, and therefore, a larger sample size could be considered in future research. It was assumed that the time interval between depression and the onset of limited daily activities was I (I represents a specific time interval or lag period theoretically or empirically determined to be necessary for a causal effect to manifest). If the observed time interval was less than I, no causal effect could be identified. Therefore, it is suggested that future research extend the time interval to explore this further, if feasible.

## 5 Conclusion

Based on the analysis of 5-year longitudinal data from PPMI, our study has revealed a positive causal relationship between RBD and depressive symptoms in Parkinson's patients. Furthermore, we have found that ADLs serve as a partial mediator in the relationship between RBD and depression. Given the interrelationship between these factors, interventions aimed at improving sleep quality or enhancing daily activities among Parkinson's patients may help reduce depression symptoms and improve their overall mental health. Further research is essential to investigate potential pathways connecting RBD and depression. Understanding these connections could enhance the quality of life for individuals with PD by breaking the potential vicious cycle between RBD and depression.

## Data availability statement

The original contributions presented in the study are included in the article/supplementary material, further inquiries can be directed to the corresponding authors.

## Author contributions

QW: Writing – original draft, Software, Data curation, Conceptualization. SZ: Writing – review & editing, Methodology, Supervision. BJ: Writing – review & editing, Supervision, Investigation. YS: Writing – review & editing, Software, Formal analysis, Data curation. WQ: Writing – review & editing, Methodology, Investigation. ZZ: Writing – review & editing, Supervision, Conceptualization. HZ: Writing – review & editing, Conceptualization.
